# Risk of upper urinary tract urothelial carcinoma after primary non‐muscle‐invasive urinary bladder cancer: A nationwide population‐based cohort study

**DOI:** 10.1002/bco2.70021

**Published:** 2025-05-05

**Authors:** Christel Häggström, Oskar Hagberg, Lars Holmberg, Abolfazl Hosseini, Tomas Jerlström, Viveka Ströck, Karin Söderkvist, Anders Ullén, Fredrik Liedberg, Staffan Jahnson, Firas Aljabery

**Affiliations:** ^1^ Department of Surgical Sciences Uppsala University Uppsala Sweden; ^2^ Northern Registry Centre, Department of Diagnostics and Intervention, Oncology Umeå University Umeå Sweden; ^3^ Institution of Translational Medicine Lund University Malmö Sweden; ^4^ School of Cancer and Pharmaceutical Sciences King's College London London UK; ^5^ Department of Molecular Medicine and Surgery Karolinska Institutet Stockholm Sweden; ^6^ Department of Urology, School of Medical Sciences, Faculty of Medicine and Health Örebro University Örebro Sweden; ^7^ Department of Urology, Sahlgrenska University Hospital and Institute of Clinical Sciences, Sahlgrenska Academy University of Gothenburg Gothenburg Sweden; ^8^ Department of Diagnostics and Intervention Umeå University Umeå Sweden; ^9^ Department of Oncology‐Pathology Karolinska Institutet Stockholm Sweden; ^10^ Department of Pelvic Cancer, Genitourinary Oncology and Urology Unit Karolinska University Hospital Stockholm Sweden; ^11^ Department of Urology Skåne University Hospital Malmö Sweden; ^12^ Department of Urology in Östergötland, and Department of Biomedical and Clinical Sciences Linköping University Linköping Sweden

**Keywords:** cohort study, epidemiology, register‐based, surveillance, upper urinary tract urothelial carcinoma, urinary bladder cancer

## Abstract

**Objectives:**

To investigate the risk of upper urinary tract urothelial carcinoma (UTUC) in patients with non‐muscle‐invasive bladder cancer (NMIBC), in relation to the primary NMIBC tumour risk categories, calendar time trends and intravesical Bacillus Calmette‐Guerin (BCG) treatment.

**Patient and methods:**

All patients with primary NMIBC diagnosed 1997–2019 registered in Bladder Cancer Data base Sweden (BladderBaSe) 2.0 were included in the study. Risk of UTUC was calculated by cumulative incidence proportion using competing risk analysis. Associations with risk of UTUC by tumour stage category, calendar time, and intravesical BCG treatment was estimated by hazard ratios from multivariable Cox regression analyses.

**Results:**

Of 36 038 NMIBC patients, 537 (1.5%) were diagnosed with UTUC during a mean time of 7 years in follow‐up. The risk of UTUC within 10 years from NMIBC diagnosis was 1.7% (95% 1.6–1.9) with highest estimates for TaG3/CIS. Stage T1 and TaG3/CIS, as compared with TaG1–2 was associated to risk, with stronger associations during later calendar times. Within high‐risk NMIBC patients (CIS/TaG3/T1), intravesical BCG treatment was associated with higher risk of UTUC.

**Conclusions:**

This large study of more than 36 000 patients with NMIBC found 1.7% (95% 1.6–1.9) risk of UTUC within 10 years of diagnosis. Differences by tumour stage category indicate the need for refined studies accounting for tumour characteristics, location in the bladder and given treatment to optimise follow‐up routines in NMIBC.

## 1 INTRODUCTION

According to the European Association of Urology (EAU) guidelines, there is a weak evidence base underlying recommendations for surveillance for upper urothelial tract carcinoma (UTUC) in patients diagnosed with non‐muscle‐invasive bladder cancer (NMIBC).^1^, ^2^, ^3^ Previous studies report a wide range of risk estimates for UTUC after NMIBC diagnosis,^4^, ^5^, ^6^, ^7^, ^8^, ^9^ the two largest studies are based on the SEER database, one reporting a 10‐year risk of 0.7% (1988–2003)^10^ and the second a risk of 0.06% (2004–2014).^11^ In 402 NMIBC patients treated with transurethral resection (TUR) followed by intravesical installations with Bacillus Calmette‐Guerin (BCG), a 7.6% risk of UTUC was observed within 10 years,^12^ and in 307 very high‐risk NMIBC patients treated with adjuvant intravesical BCG instillations, monitored for median 12 years, 25% was diagnosed with UTUC.^13^ In these previous studies, precision of the risk estimates and detailed data of risk separated for tumour stage category over follow‐up are scarce. Using regression methods, two previous reports have elucidated associations between tumour stages and risk of UTUC with inconsistent results.^4^, ^10^

Hence, larger and up‐to‐date studies are needed to provide precise estimates of the risk of UTUC after NMIBC diagnosis, considering tumour stage category, follow‐up time and possible calendar time trends. Additionally, results should account for possible confounders to further study tumour stages and the association to risk of UTUC, and within high‐risk NMIBC patients' treatment by intravesical BCG instillation and the relation to risk of UTUC need to be further evaluated.

The aim of the study was to investigate patients with primary NMIBC and their risk of UTUC, in total and separated by tumour risk categories and calendar periods, and to investigate associations with tumour risk categories, and the use of intravesical BCG treatment in high‐risk NMIBC patients, on the risk of UTUC, using data from the nationwide population‐based Swedish research database BladderBaSe 2.0.

## 2 PATIENTS AND METHODS

### 2.1 Study population selection and variable definitions

BladderBaSe 2.0 includes information about all patients with newly diagnosed NMIBC in Sweden based on data from the Swedish National Register for Urinary Bladder Cancer (SNRUBC), including patient and tumour characteristics as well as information on primary management. In addition, BladderBaSe 2.0 also includes data from the Patient Register, the Causes of Death Register, the Cancer Register and other social and demographic registers.^14^ The BladderBaSe research project was approved by The Research Ethics Board at Uppsala University, Sweden (year of approval and diary number: 2015‐277, 2019‐03574, 2020‐05123, and 2022‐01747‐02).

We selected all patients with primary NMIBC from 1997 to 2019 to the current study. We excluded patients diagnosed with UTUC prior to, or within 6 months after diagnosis of NMIBC, and patients with follow‐up less than 6 months. Time to the first diagnosis of UTUC was defined as date of tumour in the ureter or in the kidney pelvis registered in the Cancer Register using the ICD 7 codes 1802 and 1811, respectively, or ICD 10 codes C659 and C669, respectively. There was no information about the diagnostic procedures used in each individual patient. From 2013 and onwards the Swedish guidelines recommended CTU for follow‐up of upper urinary tract every year in TaG3/CIS and T1, whereas in TaG1–G2, such follow‐up was recommended in case of multiple primary tumours or multiple recurrences. Before 2013 no firm recommendations for upper tract surveillance were given for NMIBC.

We used information on intravesical BCG treatment, tumour stage subgroups and tumour grade, the latter classified according to WHO1973 from 1997 to 2002 and according to WHO1999 from 2003 and onwards and expressed as Grade 1 (G1), Grade 2 (G2) or Grade 3 (G3) registered in the SNRUBC. Papillary urothelial neoplasms with low malignant potential (PUNLMP) were analysed together with G1 tumours. Stage and grade information were categorised as Ta Grade 1–2 (TaG1–2), T1, and Ta Grade 3 (TaG3) or carcinoma in situ (CIS), these latter tumours were grouped together as TaG3 and primary CIS tumours have similar characteristics being high‐grade tumours without infiltration. Concomitant CIS together with TaG1–2, TaG3 or T1 was not registered routinely, and patients registered with CIS had only primary CIS. TaG1–G2 were used as reference group. In a sensitivity analyses, we analysed tumour stage category in categories deducted from risk‐stratification in clinical guidelines^2^: TaG1 (reference), TaG2 and CIS/TaG3/T1 grouped together. We nominated the group CIS/TaG3/T1 as high‐risk NMIBC and analysed the separate tumour categories in a subgroup analysis using T1 as reference.

### 2.2 Statistical methods

Descriptive data of proportions patient and tumour characteristics were displayed for patients included in the study population, separated by the tumour stage category. Time at follow‐up started 6 months (183 days) after NMIBC diagnosis and ended at date of diagnosis of UTUC, death of any cause, emigration or end of follow‐up 31 December 2019, whatever happened first. Risk of UTUC was assessed on an absolute scale by cumulative incidence proportions estimated by the Fine‐Gray competing risk model where death from any cause was considered as competing risk. Risks were estimated for UTUC, in total, as well separated for tumour stage categories, and in separate calendar time categories.

Associations between the different tumour stage categories and risk of UTUC was assessed by hazard ratios (HRs) based on multivariable Cox proportional hazard regression models. In these models, patients were censored at date of death of any cause or at end of follow‐up 31 December 2019, whatever happened first. Covariates included in the analyses were age at NMIBC diagnosis (72 years or less/more than 72 years), gender, calendar year of NMIBC diagnosis (1997–2001/2002–2006/2007–2011/2012–2016/2017–2019), marital status (married/not married), education level (primary school, middle or high school and university or similar) and comorbidity by Charlson comorbidity index (CCI), calculated from diagnoses recorded in the patient register up to 10 years prior to the NMIBC diagnosis and expressed as no comorbidity, mild comorbidity, intermediate comorbidity and severe comorbidity.^14^ In addition, we investigated possible effects of calendar time trends and calculated associations between tumour stage categories and risk of UTUC in separate calendar times categories. Furthermore, we investigated the association between intravesical BCG instillation and risk of UTUC within the high‐risk NMIBC subgroup using a Cox proportional hazard regression analysis adjusted for sex, age at NMIBC diagnosis, marital status, educational level, CCI, calendar year of NMIBC diagnosis and tumour stages (in three categories: T1, TaG3 and CIS). All statistical analyses were performed using R version 4.3.0.

## 3 RESULTS

The study population included 36 038 patients with primary NMIBC, of those 21 601 (60%) was diagnosed with TaG1–2, 10820 (30%) with T1 and 3617 (10%) with TaG3/CIS (Figure S1 and Table 1). The median age at diagnosis was 72 years (interquartile range 65–79 years) and during a mean time in follow‐up of 7 years (standard deviation 5 years) 537 (1.5%) patients were diagnosed with UTUC and 16 302 (45%) died from any cause.

**TABLE 1:** Baseline characteristics in patients with non‐muscle invasive bladder cancer (NMIBC) diagnosed in Sweden 1997–2019.

*n* (%)	TaG1–2	T1	TaG3/CIS	Total
*N* total	21 601	10 820	3617	36 038
Sex				
Male	16 082 (74.5)	8435 (78.0)	2989 (82.6)	27 506 (76.3)
Female	5519 (25.5)	2385 (22.0)	628 (17.4)	8532 (23.7)
Age at NMIBC diagnosis				
At most 72	11 504 (53.3)	4933 (45.6)	1648 (45.6)	18 085 (50.2)
At least 73	10 097 (46.7)	5887 (54.4)	1969 (54.4)	17 953 (49.8)
Married				
Yes	12 698 (58.8)	6327 (58.5)	2230 (61.7)	21 255 (59.0)
No	8879 (41.2)	4485 (41.5)	1384 (38.3)	14 748 (41.0)
Missing	24	8	3	35
Educational level				
Low	8703 (41.2)	4606 (43.5)	1407 (39.6)	14 716 (41.7)
Middle	8137 (38.5)	4055 (38.3)	1400 (39.4)	13 592 (38.6)
High	4279 (20.3)	1917 (18.1)	744 (21.0)	6940 (19.7)
Missing	482	242	66	790
Charlson comorbidity index				
No comorbidity (0)	12 205 (56.5)	5894 (54.5)	1954 (54.0)	20 053 (55.6)
Mild comorbidity (1)	3453 (16.0)	1864 (17.2)	634 (17.5)	5951 (16.5)
Intermediate comorbidity (2)	3438 (15.9)	1739 (16.1)	599 (16.6)	5776 (16.0)
Severe comorbidity (>2)	2505 (11.6)	1323 (12.2)	430 (11.9)	4258 (11.8)
Tumour grade				
G1/LMP	11 770(55.1)	438(4.1)	61 (1.8)	12 269 (34.7)
G2	9607 (44.9)	4005 (37.5)	88 (2.7)	13 700 (38.7)
G3	0 (0.0)	6231 (58.4)	3163 (95.5)	9394 (26.6)
Missing	224	146	305	675
Year of NMIBC diagnosis				
1997–2001	3900 (18.1)	1956 (18.1)	519 (14.3)	6375 (17.7)
2002–2006	4165 (19.3)	2042 (18.9)	615 (17.0)	6822 (18.9)
2007–2011	4795 (22.2)	2377 (22.0)	783 (21.6)	7955 (22.1)
2012–2016	5599 (25.9)	2854 (26.4)	1062 (29.4)	9515 (26.4)
2017–2019	3142 (14.5)	1591 (14.7)	638 (17.6)	5371 (14.9)

**FIGURE 1:**
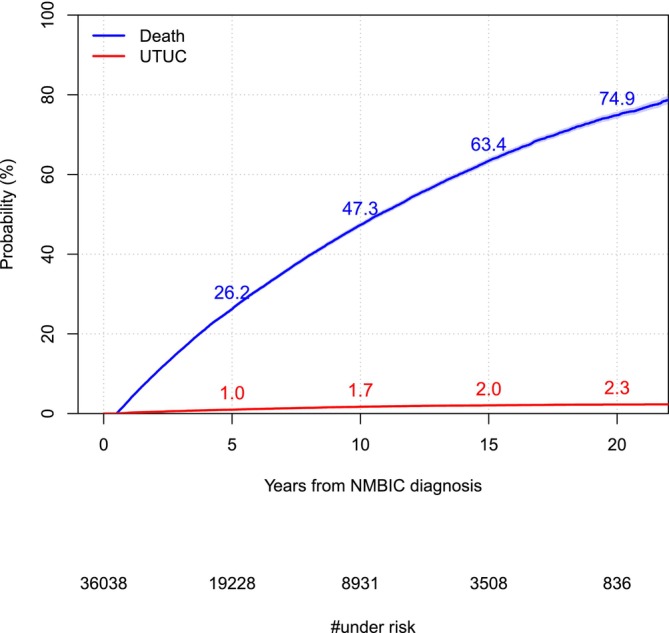
Risk of risk UTUC and death from any cause in the study population of 36 038 patients at 5, 10, 15 and 20 years after NMIBC diagnosis, estimated by cumulative incidence proportions accounting for competing risks.

**FIGURE 2:**
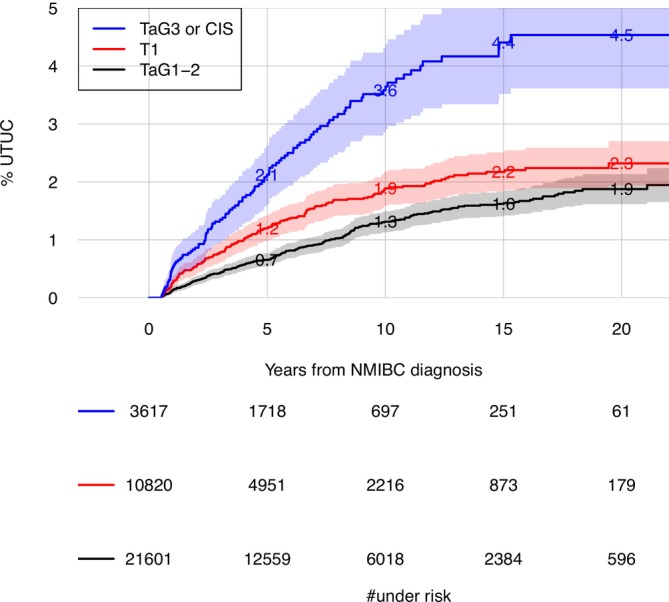
Risk of UTUC in patients with non‐muscle invasive bladder cancer (NMIBC) stratified by tumour stage category TaG1–2, T1 and TaG3 or carcinoma in situ (CIS), estimated by cumulative incidence proportions accounting for competing risks.

The risk of UTUC increased steadily over time and in all tumour stage categories but with lower rates after around 10 years (Figures 1 and 2). The risk at 10 years after NMIBC diagnosis of UTUC in the full study population was 1.7% (95% confidence interval [CI] [1.6, 1.9]) (Figure 1). Separated into tumour stage categories, the risk for TaG1–2 was 1.3% (95% CI [1.1, 1.5]) for T1 1.9% (95% CI [1.6, 2.2]) and for TaG3/CIS 3.6% (95% CI [2.9–4.3]) (Figure 2 and Table S1). At 20 years from the diagnosis of NMIBC, the risk of UTUC was 2.3% (95% CI 2.0–2.5) for the full study population, and in the tumour stage categories TaG1–2, T1 and TaG3/CIS, these risks were 1.9% (95% CI [1.6, 2.1]), 2.3% (95% CI [1.9, 2.7]) and 4.5% (95% CI [3.6, 5.5]), respectively (Figures 1 and 2, and Table S1). The risk estimates of UTUC in calendar time categories showed higher estimates in later calendar time categories (Figure S2).

Stage T1 was associated to increased risk of UTUC, multivariate adjusted HR 1.72 (95% CI [1.41, 2.09]) and TaG3/CIS, HR 2.98 (95% [CI 2.37, 3.75]), as compared with TaG1–2 (Table 2). Analyses of possible interaction effect of calendar time categories for tumour stage category indicated variations for TaG3/CIS and association to risk of UTUC across calendar times, with a tendency of higher estimates in the later calendar times. Similar tendencies but with less pronounced variations were also observed in the T1 group (Table S2).

**TABLE 2:** Associations between tumour stage category and risk of UTUC in patients with non‐muscle invasive bladder cancer (NMIBC) diagnosed in Sweden 1997–2019.

Tumour stage category	Number of patients	HR^a^	95% CI^a^	HR^b^	95% CI^b^
TaG1–2	21 601	1, ref	—	1, ref	—
T1	10 820	1.70	1.40–2.06	1.72	1.41–2.09
TaG3/CIS	3617	3.03	2.42–3.81	2.98	2.37–3.75

Abbreviations: CI, confidence interval; HR, hazard ratio; ref, reference.

^a^ HR from unadjusted Cox proportional hazards regression models.

^b^ HR from Cox proportional hazards regression models adjusted for sex, age at UBC diagnosis (2 categories), married (yes/no), educational level (3 categories), CCI (4 categories) and calendar year of UBC diagnosis (5 categories).

In sensitivity analyses of tumour stage category in categories deducted from risk stratification in clinical guidelines, we found associations to increased risk of UTUC for TaG2 and CIS/TaG3/T1 (Table S3). Furthermore, in high‐risk NMIBC, we found associations to increased risk of UTUC for TaG3 and CIS (Table S4). Within the subgroup of high‐risk NMIBC, 43% of the patients were treated with intravesical BCG, where intravesical BCG was associated with the risk of UTUC, corresponding to an HR of 1.33 (95% CI [1.03, 1.70]).

## 4 DISCUSSION

In patients diagnosed with primary NMIBC, the risk of developing UTUC within 10 years was 1.7% (95% 1.6–1.9) and 2.3% (95% 2.0–2.5) within 20 years. Within separate tumour stage categories, the risk of UTUC within 10 years varied from 1.3% to 3.6% and from 1.9% to 4.5% within 20 years. Using multivariate regression models, we found an association to risk of UTUC with T1 and TaG3/CIS as compared with TaG1–2. This association between tumour risk category and risk was more pronounced in later calendar years. Furthermore, in a subgroup analysis of high‐risk NMIBC, treatment with intravesical BCG was associated with increased risk of UTUC.

The advantages of the present study are the large, nationwide and population‐based study population with a long follow‐up time with virtually no losses to follow‐up, and the use of the mandatory National Cancer Registry for capturing the UTUC diagnoses. We calculated risk on an absolute scale by use of cumulative incidence proportions by competing risk analysis, as in the present population the considerable risk of death from other causes makes a competing risk analysis essential for reliable measures.^15^ We also, in contrast to many studies, report on the statistical precision of the risk estimates with CIs. Furthermore, the large size of the study allows for solid regression analysis such as variations in calendar times risk of UTUC. A limitation is lacking information about stage and grade of the UTUC, which could be useful for further and refined analyses. The study also lacks some information about NMIBC characteristics, such as tumour size and multiplicity, location near ureteral orifices and presence of vesicoureteral reflux. Finally, our study lacks information about potential changes in the UTUC diagnostic procedures over time.

Our results for the 10‐year risk of UTUC are in the in the range of other studies, varying from 0.06% to 4%.^4^, ^5^, ^6^, ^7^, ^8^, ^9^, ^10^, ^11^ A head‐to‐head comparison of our results with the previous studies is difficult. In most studies, we lack information on CIs of their risk estimates. Our analyses are also the only accounting for competing risk of death. Follow‐up routines may vary between studies with different capacity to identify metachronous UTUC. The results by tumour stage category indicate that the baseline characteristics of the cohorts are of paramount importance for the overall outcome. Nevertheless, our data together with the bulk of evidence^4^, ^5^, ^6^, ^7^, ^8^, ^9^, ^10^, ^11^, ^12^ point to a 10‐year risk well below 4% but higher than 1% in a population‐based cohort.

The risk of UTUC differed for the different tumour stage categories, with lowest estimates for TaG1–2 and highest for TaG3/CIS category. Our results are also underlined by the subgroup analysis where CIS and TaG3 had increased risk of UTUC as compared with T1. Most studies have found tumour grade and T category to be important factors for higher risk of UTUC after NMIBC,^4^, ^5^, ^6^, ^7^, ^8^, ^9^, ^10^, ^11^ indicating the clinical relevance of these factors to design an upper urinary tract follow‐up programme after NMIBC.

To our knowledge, only two studies have investigated the associations between categories of stage and/or grade and the risk of UTUC using multivariate regression models. Wright et al.^10^ reported an increased risk of UTUC for Grades 2 and 3 tumours, as well as a borderline increased risk of UTUC for CIS as compared with Ta tumours. In contrast, Millán‐Rodríguez et al.^4^ reported no statistically significant estimates for T stage or grade in an exploratory study after multivariable adjustment. In our study, associations to risk of UTUC based on multivariate analysis indicate that the stages T1 and TaG3/CIS as compared with TaG1–2 was associated to risk also after adjusting for age, sex, comorbidity or socio‐economic factors. Other studies have suggested that multiplicity,^4^ tumour location near the urethral orifice,^6^, ^8^, ^10^ tumour recurrence in the bladder^6^, ^9^ or timing of bladder cancer recurrences^9^ could be associated to risk.

We found a higher risk at later calendar times. Possible reasons for this are not straightforward, but our analysis of potential interaction effect of calendar time categories for tumour stage category and risk of UTUC with a tendency of higher estimates in the later calendar times for T1 and TaG3/CIS, indicate an effect of more accurate diagnosis in later years using ureteroscopy and voided urine cytology and a higher awareness of the importance of adequate registration of UTUC. This may follow from those more active recommendations for surveillance of the upper urinary tract in patients with NMIBC has evolved over time and that their implementation has been successful.^1^, ^3^, ^16^

In high‐risk NMIBC, treatment with BCG was associated with increased risk of UTUC. These results are in accordance with previous studies.^12^, ^17^ Whether this is due to intravesical BCG treatment per se or that intravesical BCG treatment is a proxy for more aggressive tumours need to be further studied. In our analysis, we adjusted for tumour stage; however, residual confounding may remain, and future studies with more granular data regarding tumour characteristics would be valuable. Ureteric stents inserted at TUR might lead to increased risk of UTUC in NMIBC as observed by Hupe et al.,^18^ probably due to tumour cell reflux from the bladder to the upper urinary tract. However, our study lacks information about the use of stents in conjunction with TUR.

## 5 CONCLUSIONS

In this large population‐based cohort, patients with NMIBC have 1.7% (95% 1.6–1.9) risk of UTUC within 10 years of diagnosis. The risk differed by tumour stage category, which indicates that follow‐up routines need to be optimised for maximum cost–benefit for this patient group. We need—preferably prospective—studies accounting for NMIBC diagnostic stages, tumour location, multifocality and given treatment to weigh pros and cons of different management routines to provide evidence to design rational follow‐up routines and patient information.

## AUTHOR CONTRIBUTIONS

C. H.: conceptualization, data curation, methodology, investigation, resources and writing the original draft. O.H.: conceptualization, formal analysis, data curation, methodology, investigation and writing the original draft. L. H and F. L.: conceptualization, data curation, methodology, investigation, funding acquisition, resources, supervision and writing the original draft. A. H., T. J., V. S., K. S. and A.U.: data curation and writing review and editing. S. J. and F. A.: conceptualization, data curation, methodology, investigation, resources, supervision and writing the original draft.

## CONFLICT OF INTEREST STATEMENT

None declared.

## Supporting information


**Table S1.** Risk of UTUC and death of any cause at 2, 5, 10, 15, and 20 years after NMIBC diagnosis, separated by tumour stage category, estimated by cumulative incidence proportions accounting for competing risks.
**Table S2.** Associations between tumour stage category and risk of UTUC in calendar time categories of NMIBC diagnosis
**Table S3.** Sensitivity analysis of the associations between tumour stage categories related to clinical risk‐stratification in guidelines and risk of UTUC in patients with non‐muscle invasive bladder cancer (NMIBC) diagnosed in Sweden 1997–2019.
**Table S4.** Subgroup analysis of 14 437 high risk NMIBC patients (CIS, TaG3, or T1) of the associations and risk of UTUC in Sweden 1997–2019.
**Figure S1.** Flowchart‐diagram describing the study population selection
**Figure S2.** Risk of UTUC in patients with non‐muscle invasive bladder cancer (NMIBC) in categories of calendar year of NMIBC diagnosis, estimated by cumulative incidence proportions accounting for competing risks.
