# Supplementary material for: Risk of upper urinary tract urothelial carcinoma after primary non‐muscle‐invasive urinary bladder cancer: A nationwide population‐based cohort study
Source: BJUI Compass. 2025 May 5;6(5):e70021. doi: 10.1002/bco2.70021 (PMC12050951; doi:10.1002/bco2.70021)

**Supplemental Table 1:** Risk of UTUC and death of any cause at 2, 5, 10, 15, and 20 years after NMIBC diagnosis, separated by tumour stage category, estimated by cumulative incidence proportions accounting for competing risks.

| Years in follow up | Tumour stage category | UTUC diagnosis | Death |
| --- | --- | --- | --- |
| 2 | TaG1-2 | 0.29 (0.22-0.37) | 6.90 (6.56-7.25) |
|  | T1 | 0.54 (0.40-0.68) | 16.33 (15.61-17.04) |
|  | TaG3/CIS | 0.86 (0.56-1.17) | 8.94 (7.98-9.89) |
| 5 | TaG1-2 | 0.65 (0.54-0.77) | 21.02 (20.43-21.60) |
|  | T1 | 1.20 (0.98-1.41) | 35.92 (34.95-36.90) |
|  | TaG3/CIS | 2.12 (1.61-2.63) | 28.47 (26.84-30.10) |
| 10 | TaG1-2 | 1.31 (1.13-1.49) | 42.24 (41.43-43.04) |
|  | T1 | 1.89 (1.59-2.18) | 56.60 (55.47-57.73) |
|  | TaG3/CIS | 3.58 (2.86-4.31) | 50.32 (48.24-52.40) |
| 15 | TaG1-2 | 1.62 (1.41-1.83) | 59.61 (58.66-60.55) |
|  | T1 | 2.17 (1.84-2.51) | 70.57 (69.35-71.79) |
|  | TaG3/CIS | 4.41 (3.52-5.29) | 65.15 (62.74-67.56) |
| 20 | TaG1-2 | 1.87 (1.62-2.13) | 71.36 (70.22-72.50) |
|  | T1 | 2.32 (1.94-2.70) | 81.34 (79.95-82.74) |
|  | TaG3/CIS | 4.53 (3.61-5.46) | 77.15 (74.36-79.94) |

**Supplemental Table 2.** Associations between tumour stage category and risk of UTUC in calendar time categories of NMIBC diagnosis

|  | **1997-2001** | **2002-2006** | **2007-2011** | **2012-2016** | **2017-2019** |
| --- | --- | --- | --- | --- | --- |
| **Tumour stage category** | **HR (95% CI)** | **HR (95% CI)** | **HR (95% CI)** | **HR (95% CI)** | **HR (95% CI)** |
| **TaG1-2** | 1,ref | 1,ref | 1,ref | 1,ref | 1,ref |
| **T1** | 1.47 (0.95-2.27) | 1.30 (0.87-1.94) | 2.19 (1.49-3.21) | 2.04 (1.37-3.03) | 1.69 (0.73-3.94) |
| **TaG3/CIS** | 1.49 (0.74-3.03) | 1.99 (1.18-3.34) | 4.68 (3.09-7.07) | 3.66 (2.37-5.65) | 2.53 (0.94-6.78) |

HR: Hazard ratio, CI: Confidence interval, ref: reference

HR from Cox proportional hazards regression models adjusted for sex, age at UBC diagnosis (2 categories), married (yes/no), educational level (3 categories), and CCI (4 categories).

**Supplement Table 3:** Sensitivity analysis of the associations between tumour stage categories related to clinical risk-stratification in guidelines and risk of UTUC in patients with non-muscle invasive bladder cancer (NMIBC) diagnosed in Sweden 1997-2019.

| Tumour stage  category | Number of patients | HR* | 95% CI* | HR** | 95% CI** |
| --- | --- | --- | --- | --- | --- |
| TaG1 | 11994 | 1, ref | - | 1,ref |  |
| TaG2 | 9607 | 1.91 | 1.49-2.47 | 1.99 | 1.54-2.56 |
| CIS/TaG3/T1 | 14437 | 2.83 | 2.26-3.55 | 2.90 | 2.30-3.64 |

HR: Hazard ratio, CI: Confidence interval, ref: reference

*HR from unadjusted Cox proportional hazards regression models

**HR from Cox proportional hazards regression models adjusted for sex, age at UBC diagnosis (2 categories), married (yes/no), educational level (3 categories), CCI (4 categories), calendar year of UBC diagnosis (5 categories)

**Supplement Table 4:** Subgroup analysis of 14437 high risk NMIBC patients (CIS, TaG3, or T1) of the associations and risk of UTUC in Sweden 1997-2019.

| Tumour stage | Number of patients | HR* | 95% CI* | HR** | 95% CI** |
| --- | --- | --- | --- | --- | --- |
| T1 | 10820 | 1, ref | - | 1, ref |  |
| TaG3 | 2289 | 1.51 | 1.11-2.04 | 1.41 | 1.04-1.92 |
| CIS | 1328 | 2.19 | 1.61-2.98 | 2.19 | 1.61-2.99 |

HR: Hazard ratio, CI: Confidence interval, ref: reference

*HR from unadjusted Cox proportional hazards regression models

**HR from Cox proportional hazards regression models adjusted for sex, age at UBC diagnosis (2 categories), married (yes/no), educational level (3 categories), CCI (4 categories), calendar year of UBC diagnosis (5 categories)

**Supplement Figure 1.** Flowchart-diagram describing the study population selection

**Supplement Figure 2.** Risk of UTUC in patients with non-muscle invasive bladder cancer (NMIBC) in categories of calendar year of NMIBC diagnosis, estimated by cumulative incidence proportions accounting for competing risks.
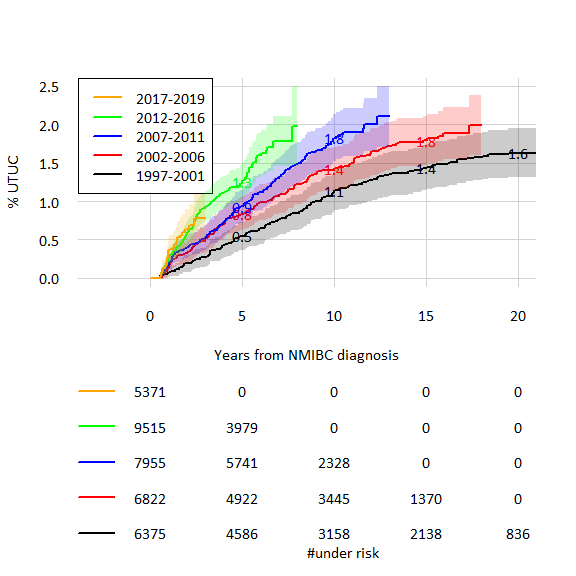

Supplement: Supplementary file 1 — Table S1. Risk of UTUC and death of any cause at 2, 5, 10, 15, and 20 years after NMIBC diagnosis, separated by tumour stage category, estimated by cumulative incidence proportions accounting for competing risks. Table S2. Associations between tumour stage category and risk of UTUC in calendar time categories of NMIBC diagnosis Table S3. Sensitivity analysis of the associations between tumour stage categories related to clinical risk‐stratification in guidelines and risk of UTUC in patients with non‐muscle invasive bladder cancer (NMIBC) diagnosed in Sweden 1997–2019. Table S4. Subgroup analysis of 14 437 high risk NMIBC patients (CIS, TaG3, or T1) of the associations and risk of UTUC in Sweden 1997–2019. Figure S1. Flowchart‐diagram describing the study population selection Figure S2. Risk of UTUC in patients with non‐muscle invasive bladder cancer (NMIBC) in categories of calendar year of NMIBC diagnosis, estimated by cumulative incidence proportions accounting for competing risks. [file BCO2-6-e70021-s001.docx]
